# Mesenchymal Stem Cells Overexpressing ACE2 Favorably Ameliorate LPS-Induced Inflammatory Injury in Mammary Epithelial Cells

**DOI:** 10.3389/fimmu.2021.796744

**Published:** 2022-01-14

**Authors:** Shuping Yan, Pingsheng Ye, Muhammad Tahir Aleem, Xi Chen, Nana Xie, Yuanshu Zhang

**Affiliations:** ^1^ Key Laboratory of Animal Physiology and Biochemistry, Ministry of Agriculture, College of Veterinary Medicine, Nanjing Agricultural University, Nanjing, China; ^2^ Ministry of Education (MOE) Joint International Research Laboratory of Animal Health and Food Safety, College of Veterinary Medicine, Nanjing Agricultural University, Nanjing, China

**Keywords:** ACE2, MSCs, EpH4-Ev cells injury, IL-10/STAT3/SOCS3 signaling pathway, blood-milk barrier

## Abstract

Mesenchymal stem cells (MSCs) are capable of homing injury sites to exert anti-inflammatory as well as anti-damage effects and can be used as a vehicle for gene therapy. Angiotensin-converting enzyme 2 (ACE2) plays an important role in numerous inflammatory diseases, but fewer studies have been reported in animal mastitis. We hypothesized that MSCs overexpressing ACE2 is more effective in ameliorating lipopolysaccharide (LPS)-induced inflammatory injury in mammary epithelial cells compared to MSCs alone. The results showed that MSC-ACE2 inhibited the LPS induction by upregulation of TNF-α, IL-Iβ, IL-6, and iNOS mRNA expression levels in EpH4-Ev cells compared with MSCs. Furthermore, results showed that both MSC and MSC-ACE2 were significantly activated IL-10/STAT3/SOCS3 signaling pathway as well as inhibited TLR4/NF-κB and MAPK signaling pathways, but MSC-ACE2 had more significant effects. Meanwhile, MSC-ACE2 promoted the expression of proliferation-associated proteins and inhibited the expression of the apoptosis-associated proteins in EpH4-Ev cells. In addition, MSC and MSC-ACE2 reversed the LPS-induced downregulation expression levels of the tight junction proteins in mammary epithelial cells, indicating that both MSC as well as MSC-ACE2 could promote blood-milk barrier repair, and MSC-ACE2 was more effective. These results suggested that MSCs overexpressing ACE2 were more anti-inflammatory as well as anti-injurious action into LPS-induced inflammatory injury in the EpH4-Ev cells. Thus, MSCs overexpressing ACE2 is expected to serve as a potential strategy for mastitis treatment.

## Introduction

Mastitis is an inflammatory disease caused by a variety of pathogens, the highest incidence of which is *Staphylococcus aureus*, *E. coli*, *streptococcus uberis*, etc. (1). Mastitis in dairy cattle causes huge economic losses to the livestock industry and there is no particularly effective treatment. Lipopolysaccharide (LPS), a major component of the cell wall of Gram-negative bacteria, is often used to model mastitis. LPS induces an inflammatory response by activating the toll receptor-4 (TLR4)/nuclear factor-κB (NF-κB) signaling pathway, which in turn promotes the secretion of tumor necrosis factor-α (TNF-α), interleukin-Iβ (IL-Iβ), and interleukin-6 (IL-6) (2–4). Therefore, in this study, we will use LPS to establish a typical model of inflammation in EpH4-Ev cells (Mouse mammary epithelial cells).

ACE2, a key enzyme of the renin-angiotensin system (RAS), plays an important role in many inflammatory diseases and has strong anti-inflammatory action as well as anti-damage effects (5). Li et al. discovered that ACE2/Ang1-7/Mas prohibited LPS-prompted apoptosis of microvascular pulmonary endothelial cells by hindering JNK/NF-κB pathway (6). Wang et al. reported that ACE2/Ang1-7/Mas might considerably restrain pancreatitis and decrease the discharge of inflammatory cytokines (7). The surveys of ACE2 in the mammary gland centralized on breast cancer in humans (8, 9), but some asserted the ACE2 and its impact on inflammatory reaction in mammary tissue. Our previous study showed that LPS treatment of EpH4-Ev cells for 9 h showed a trend of ACE2 first increasing and then decreasing, and was also involved in LPS-induced injury to EpH4-Ev cells (10).

Mesenchymal stem cells (MSCs) are currently gaining attention as the most promising therapeutic strategy for the medication of inflammatory diseases. It has been proposed that MSCs can migrate to the site of inflammatory injury and modulate the function of multiple subtypes of immune cells through direct contact, paracrine exosomes, cytokines and transfer of mitochondria to reduce the release of oxidative stress molecules, as well as inflammatory factors (11–13). However, the mechanism of action is still unclear. So, our first aim in this study is to establish MSCs lines that consistently over-express ACE2 utilizing mouse vectors (translation elongation factor 1α (EF1α) and promoter-dependent lentiviral). Secondly, to estimate the role as well as the mechanism of ACE2 gene-modified MSCs in suppressing LPS-induced inflammation and repairing EpH4-Ev cells using by *in vitro* experimental approach. These studies support a base for continued examining of the valuable impacts of ACE2-modified MSCs in animal models of supra-mammary inflammation.

## Materials and Methods

### MSCs Transduction by Lentiviral Vectors

Rats MSCs utilized in this research were procured from the National Collection of Authenticated Cell Cultures (Shanghai, China). These MSCs were isolated from the bone marrow of Wistar rats. Consistent with expectations, these MSCs were positive for CD90 expression (Positive rate of 99.68%) and negative for CD45 and CD34 expression (Additional file 1: **Figure 1**). Additionally, these possess the potential to differentiate into osteocytes, adipocytes, and chondrocytes, as demonstrated in assays performed by the supplier.

**Figure 1:**
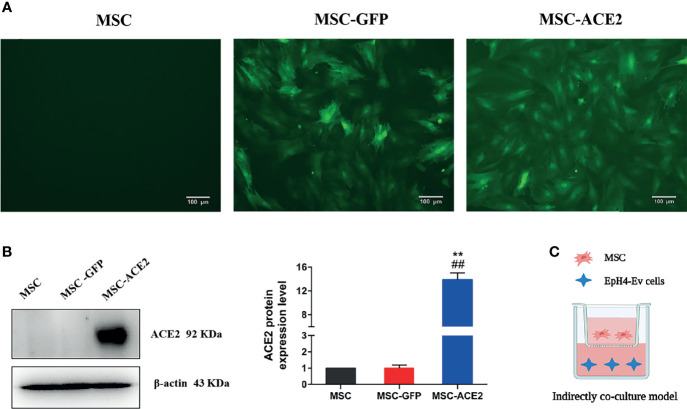
Stable genetic modification of MSCs with a lentiviral vector mediates ACE2 gene transduction. **(A)** Examples of transduced MSC-ACE2 morphology at passage 4 under phase control and fluorescence microscopy, the transduction efficiency as assessed by detecting GFP expression was as high as 100%. **(B)** The ACE2 protein expression in MSCs was assessed by Western blot. Experiments were repeated three times and data were presented as the mean ± SEM (n = 4). Approximately 12-fold higher levels were represented in the MSC-ACE2 group than the MSC and MSC-GFP groups (^**^ *P* < 0.01 vs. MSC; ^##^ *P* < 0.01 vs. MSC-GFP). **(C)** An indirect co-culture model system was utilized to examine the therapeutic impact of MSC, MSC-GFP, as well as MSC-ACE2 on resisting LPS-induced injury in EpH4-Ev cells.

Transduction of MSCs with lentiviral vectors was purchased from Shanghai Genechem Co., Ltd. (Shanghai, China). The lentivirus vector system consists of three plasmids (GV lentivirus vector series, pHelper 1.0 vector and pHelper 2.0 vector (Genechem Co., Ltd., Shanghai, China)) and cloned with the full-length coding sequence of ACE2 (NM_001130513.1, 2418 bp). Afterward, cells (293FT) were transduced thru lentiviral (expression vector) and another three wrapping plasmids, (like GV lentivirus vector series, pHelper 1.0 vector, and pHelper 2.0 vector) by using Lipofectamine 3000 (Thermo Fisher Scientific Inc., USA) to acquire the highest titer recombinant lentiviral vectors. The MSCs were transfected with carrying GFP (MSC-GFP) or both the ACE2 gene as well as GFP labeled lentivirus. After 96 h transfection, the cells have partitioned the puromycin (4 µg/mL) for additional 5 days until no cell died any longer. Afterward, the marked cells were expanded.

The expression of GFP was detected using a fluorescent microscope to evaluate the long-term transduction efficiency transduced MSCs cultured in a complete culture medium for over 30 passages. The expression of ACE2 protein was measured by Western blot. MSC-GFP and MSC-ACE2 from passages 5~10 were used in this study.

### Modeling of EpH4-Ev Cell Injury

Mouse mammary epithelial cells (EpH4-Ev) were provided by Prof. Jinfeng Miao from Nanjing Agricultural University. The method of cell culture passage was referred to Wang et al. (10). According to the results of Wang et al, 1 µg/mL of LPS concentration treated with EpH4-Ev for 9 h caused significant cell injury, so 1 µg/mL of LPS treated with EpH4-Ev for 9 h was chosen as the induction condition for the mastitis model in current study.

### Transwell and Co-Culture of EpH4-Ev and MSCs

EpH4-Ev cells were indirectly co-cultured thru stem cells (MSCs, MSC-GFP, or MSC-ACE2) in collagenous-covered pore size (0.4 µm) trans-well methods (Corning Inc., NY) (**Figure 1C**). In brief, 1 × 10^6^ EpH4-Ev cells per well were plated in 6-well culture dishes (1.5 mL) in DMEM accompanied with 10% FBS. Afterward, implanted MSCs (2 × 10^5^) in the upper chamber of the trans-well thru culture medium (750 µL) and were co-cultured with EpH4-Ev cells (37°C for 24 h, 5% CO_2_ incubator). Thereafter, the co-cultures were treated with 1 µg/mL of LPS for 9 h. The EpH4-Ev cells were cultured into the culture medium as negative controls. The EpH4-Ev cells were processed *via* LPS but absence co-cultured thru MSCs as positive controls. The EpH4-Ev cells were reaped for quantitative real-time PCR trials of inflammatory intermediaries, and utilized for Western blot.

### Detection of Ang II, Ang-(1-7) Concentrations and NAGase Activity

The Ang II and Ang-(1-7) concentrations in the cell culture supernatant was calculated *via* ELISA (Hengyuan Biotechnology Co, Shanghai, China). ELISA test was conducted according to the manufacturer’s instructions. The concentration of Ang II and Ang-(1-7) was expressed as ng/L. The activity of N-acetyl-β-D-glucosaminidase (NAGase) in the cell culture supernatant was determined according to the instructions of the kit, which was purchased from the Nanjing Jiancheng Bioengineering Institute (Nanjing, China). The level of the activity of NAGase was expressed as U/L.

### Quantitative Real-Time PCR (qRT-PCR)

The relative transcript levels of TNF-α, IL-Iβ, IL-6, IL-10 and iNOS in EpH4-Ev cells were analyzed by qRT-PCR assay. First, the cells were acquired, and the total RNA was extorted with a TRIZOL reagent kit (Vazyme, Nanjing, China) under the manufacturer’s guidance. Total RNA (2 μg) was reverse‐transcribed into cDNA through the Superscript II kit (Vazyme, Nanjing, China) according to the manufacturer’s recommendations. The β-actin gene was used as a reference gene. The primer sequences were listed in **Table S1** (Additional file 2). All samples were examined in triplicate and programmed to conduct one cycle (95°C for 30 sec) and 40 cycles (95°C for 10 sec, 60°C for 30 sec). The dissolution curves were generated according to the following procedures: 95°C/15 sec, 60°C/1 min, and 95°C/15 sec. The data were calculated according to Raw cycle thresholds (Ct) which were attained from iQ5 Sequence Detection software (Bio-Rad, California, USA) used by the relative Ct (2^-ΔΔCt^) method.

### Western Blot Analysis

The proteins (30 µg) were isolated by 10% sodium dodecyl sulfate‐polyacrylamide gel electrophoresis and transferred onto polyvinylidene fluoride (PVDF) membranes (GE Healthcare Life Science, Beijing). To block nonspecific binding, the membranes were incubated with 5% BSA for 2 h at room temperature. Washed 5 times with TBST (Tris-buffered saline solution consisting of 0.1% Tween-20 solution). Afterward the membranes were incubated with primary antibody overnight at 4°C. Again washed with TBST, and then the membranes were incubated with HRP-labeled secondary antibodies (Goat anti-rabbit IgG, ABclonal, Wuhan, China) for 2 h at room temperature. The expression of β-actin was an internal quantitative control. Again, membranes were washed by TBST and stained with Tanon™ High-sig ECL Western blot substrate kit (Pierce, Rockford, IL). The relative proteins expression levels were analyzed by Image J software. Source antibody: ACE2 (abcam, USA); TNF-α (Santa cruz, USA); Bax, Caspase‐3, Bcl2, and Caspase‐9 (ABclonal, Wuhan, China); IL-Iβ, iNOS, PCNA (Proteintech, USA); p38, p38 (phospho-T180), ERK, p-ERK (Phospho-T202), STAT3 (Bioworld, Nanjing, China); MyD88, JNK, p-JNK (phospho Thr183/Y185), p65, NF-κB-p65 (phospho Ser536) (Immunoway, USA); IL-6, IL-10, p-STAT3 (phospho Ser727), ZO-1, Claudin-1, Claudin-2 (Affinity Biosciences, USA).

### Cell Proliferation Assay

The EpH4-Ev cells were co-cultured with MSCs, MSC-GFP, or MSC-ACE2 in 96-well culture plates (Corning Inc., NY). Group settings were consistent with the description of Co-Culture. The cell proliferation assays were detected by adding 10 μL of CCK-8 solution (Beyotime, Nanjing, China) with another 2 h incubation. After which, the OD_450_ (Optical density) values were calculated by using a microplate reader (Thermo Scientific, USA).

### Statistical Analysis

All results were expressed as the mean ± SEM (standard error of the mean). LPS treatment group equated with the control group and analyzed by the Independent-Samples T-test Compared through the Means of SPASS 11.0 for Windows (StatSoft, Inc., Tulsa, USA). LPS treatment group compared with other groups and investigated using one-way ANOVA. The *P* values < 0.05 were considered statistically significant.

## Results

### ACE2 Gene Modification of MSCs

Inverted fluorescence microscope image (**Figure 1A**) showed GFP expression with fluorescence transduction efficiency of more than 95%, and still maintained such transduction efficiency for more than 30 passages. Western blot showed that the relative expression level of ACE2 protein in MSC-ACE2 group was about 12-fold higher than that in MSC and MSC-GFP groups (^**^ *P* < 0.01 *vs*. MSC; ^##^ *P* < 0.01 *vs*. MSC-GFP) (**Figure 1B**).

### MSC-ACE2 Inhibited the LPS-Induced Elevation of NAGase Activity in EpH4-Ev Cells

**Figure 2:**
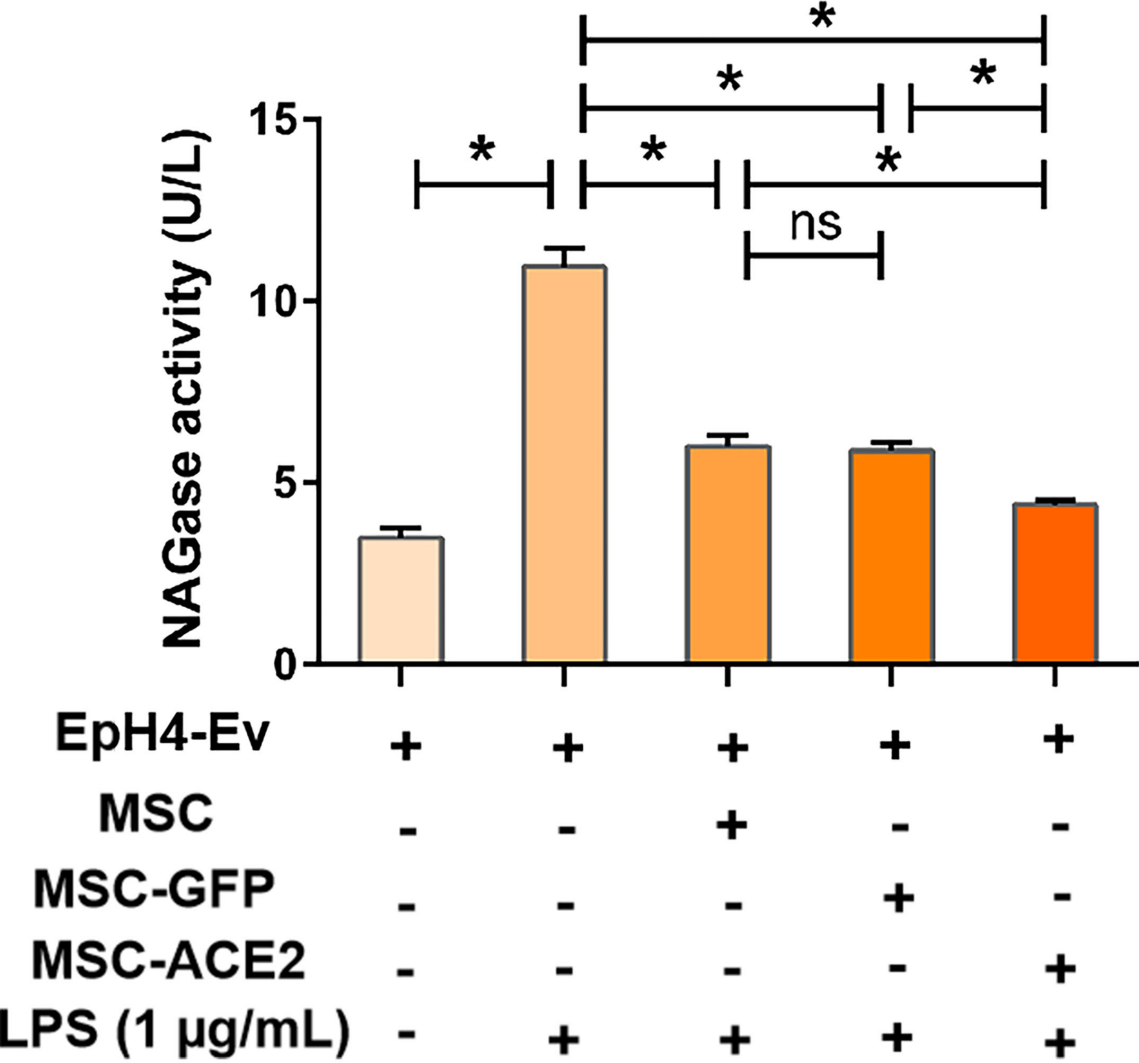
MSC-ACE2 inhibits the LPS-induced elevation of NAGase activity in EpH4-Ev cells. Detection of NAGase activity in cell culture supernatant according to kit instructions. Experiments were repeated three times and data were presented as the mean ± SEM (n = 4). *P < 0.05 (significantly different) between the indicated groups, ns, no significant difference.

The results (**Figure 2**) showed that NAGase activity in the cell culture supernatant was significantly increased by 1 μg/mL LPS treatment of EpH4-Ev cells for 9 h (^*^ *P* < 0.05 *vs*. EpH4-Ev), while NAGase activity was significantly decreased by co-culture with MSC or MSC-GFP group (^#^ *P* < 0.05 *vs*. LPS). Interestingly, MSC-ACE2 group reduced NAGase activity more strongly than MSC and MSC-GFP groups (*^$^P* < 0.05 *vs*. MSC; ^&^ *P* < 0.05 *vs*. MSC-GFP).

### MSC-ACE2 Upregulated Ang-(1-7) Levels and Downregulated Ang II Levels in EpH4-Ev Cells

**Figure 3:**
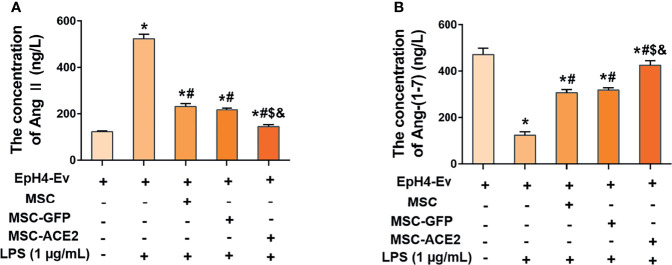
MSC-ACE2 inhibited the LPS-induced increase in Ang II concentration and decrease in Ang-(1-7) concentration in EpH4-Ev cells. **(A)** Detection of Ang II concentration in cell culture supernatants by ELISA. **(B)** Detection of Ang-(1-7) concentration in cell culture supernatants by ELISA. Experiments were repeated three times and data were presented as the mean ± SEM (n = 4). *P < 0.05 vs. EpH4-Ev; ^#^ *P* < 0.05 vs. LPS; ^$^ *P* < 0.05 vs. MSC; ^&^ *P* < 0.05 vs. MSC-GFP.

As shown in **Figure 3**, LPS treatment up-regulated Ang II levels and down-regulated Ang-(1-7) levels in EpH4-Ev cells compared with the control group (^*^ *P* < 0.05 *vs*. EpH4-Ev). However, Ang II levels were significantly downregulated and Ang-(1-7) levels were significantly upregulated in the MSC, MSC-GFP, and MSC-ACE2 groups compared with the LPS group (^#^ *P* < 0.05 *vs*. LPS). Moreover, the effect was more pronounced in the MSC-ACE2 group compared with MSC, MSC-GFP groups (*^$^P* < 0.05 *vs*. MSC; ^&^ *P* < 0.05 *vs*. MSC-GFP).

### MSC-ACE2 Downregulated the Levels of Pro-Inflammatory Mediators in EpH4-Ev Cells

**Figure 4:**
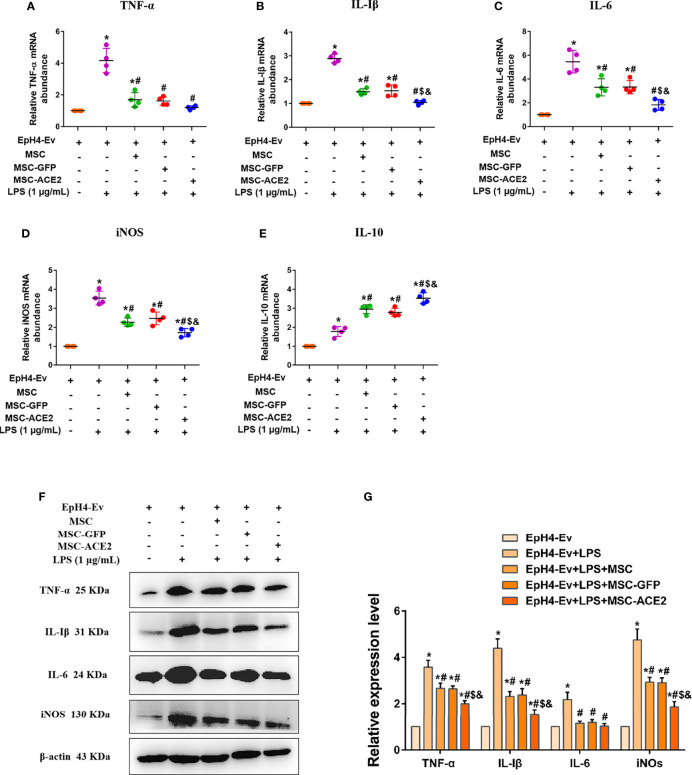
MSC-ACE2 downregulates the production of inflammatory mediators. **(A–E)** Detection of relative transcript levels of TNF-α, IL-1β, IL-6, iNOS, and IL-10 in EpH4-Ev cells by qRT-PCR. TNF-α, tumor necrosis factor-α; IL-Iβ, interleukin-Iβ; IL-6, interleukin-6; iNOS, inducible nitric oxide synthase; IL-10, interleukin-10. **(F)** Detection of relative protein expression levels of TNF-α, IL-1β, IL-6, iNOS, and IL-10 by Western blot. **(G)** Statistics of the TNF-α, IL-1β, IL-6, and iNOS Western blot results. Experiments were repeated three times and data were presented as the mean ± SEM (n = 4). *P < 0.05 vs. EpH4-Ev; ^#^ *P* < 0.05 vs. LPS; ^$^ *P* < 0.05 vs. MSC; ^&^ *P* < 0.05 vs. MSC-GFP.

Studies have reported 1 µg/mL of LPS concentration treated with EpH4-Ev cells for 9 h caused significant cell injury (10). The qPCR results (**Figures 4A–D**) showed that the transcript levels of TNF-α, IL-Iβ, IL-6, and iNOS were significantly upregulated after LPS treatment of EpH4-Ev cells (^*^ *P* < 0.05 *vs*. EpH4-Ev). However, transcript levels of these inflammatory mediators were significantly reduced after co-culture with MSC, MSC-GFP, or MSC-ACE2 group (^#^ *P* < 0.05 *vs*. LPS). Moreover, the effect was more pronounced in the MSC-ACE2 group compared with MSC, MSC-GFP groups (*^$^P* < 0.05 *vs*. MSC; ^&^ *P* < 0.05 *vs*. MSC-GFP). Consistent with expectations, Western blot results (**Figures 4F, G**) showed that the expression levels of TNF-α, IL-Iβ, IL-6 and iNOS were significantly upregulated after LPS treatment of EpH4-Ev cells (^*^ *P* < 0.05 *vs*. EpH4-Ev). However, the expression levels of these inflammatory mediators were significantly down-regulated after co-culture with MSC, MSC-GFP or MSC-ACE2 (^#^ *P* < 0.05 *vs*. LPS). Furthermore, the effect was more pronounced in the MSC-ACE2 group compared with MSC and MSC-GFP groups (*^$^P* < 0.05 *vs*. MSC; ^&^ *P* < 0.05 *vs*. MSC-GFP).

Studies also have shown that MSC secretes some cytokines and chemokines (e.g., IL-10, TGF-β, HGF, etc.) through paracrine action for the treatment of inflammation and tissue repair (11–14). In this study, IL-10 mRNA results (**Figure 4E**) showed that IL-10 levels were significantly increased after LPS stimulation of EpH4-Ev cells compared to the control group (^*^ *P* < 0.05 *vs*. EpH4-Ev). After co-culture by MSC, MSC-GFP or MSC-ACE2 group, the mRNA expression level of IL-10 was significantly increased, and the highest IL-10 expression was observed in MSC-ACE2 group (^#^ *P* < 0.05 *vs*. LPS; *^$^P* < 0.05 *vs*. MSC; ^&^ *P* < 0.05 *vs*. MSC-GFP).

### MSC-ACE2 Promotes the Proliferation of EpH4-Ev Cells and Inhibits the Apoptosis of EpH4-Ev Cells

**Figure 5:**
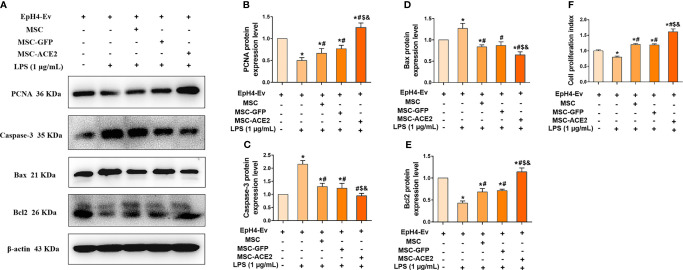
MSC-ACE2 promotes the proliferation of EpH4-Ev cells and inhibits the apoptosis of EpH4-Ev cells. **(A)** Detection of relative protein expression levels of PCNA, Caspase-3, Bax, and Bcl-2 by Western blot; **(B–E)** Statistics of the PCNA, Caspase-3, Bax, and Bcl-2 Western blot results (n = 4). **(F)** CCK8 detects EpH4-Ev cells proliferation (n = 6). Experiments were repeated three times and data were presented as the mean ± SEM. *P < 0.05 vs. EpH4-Ev; ^#^ *P* < 0.05 vs. LPS; ^$^ *P* < 0.05 vs. MSC; ^&^ *P* < 0.05 vs. MSC-GFP.

The study suggested that regulation of cell proliferation and apoptosis may be another mechanism by which MSCs exert their therapeutic effects (15). Therefore, we next examined the protein expression of PCNA, a marker of cell proliferation, and Caspase-3, Bax and Bcl2, apoptosis-related proteins by Western blot. The results (**Figure 5F**) showed that LPS treatment of EpH4-Ev cells significantly inhibited the proliferation of EpH4-Ev cells compared with the control group (^*^ *P* < 0.05 *vs*. EpH4-Ev). However, MSC, MSC-GFP or MSC-ACE2 group significantly promoted the proliferation of Eph4-Ev cells after co-culture with Eph4-Ev cells compared to the LPS-treated group (^#^ *P* < 0.05 *vs*. LPS). Moreover, the MSC-ACE2 combination treatment showed a stronger effect, significantly higher than MSC and MSC-GFP treatment alone (^$^ *P* < 0.05 *vs*. MSC; ^&^ *P* < 0.05 *vs*. MSC-GFP). Furthermore, Western blot results (**Figures 5A–E**) showed that co-culture with MSC, MSC-GFP or MSC-ACE2 significantly reversed the LPS-induced down-regulation of PCNA and Bcl2 (anti-apoptotic protein) expression levels in Eph4-Ev cells, as well as the down-regulation of pro-apoptotic protein (Bax, Caspase-3) expression levels (^#^ *P* < 0.05 *vs*. LPS). The effect was more pronounced in the MSC-ACE2 group compared with MSC and MSC-GFP groups (^$^ *P* < 0.05 *vs*. MSC; ^&^ *P* < 0.05 *vs*. MSC-GFP). These results suggested that MSC, as well as MSC-GFP, can significantly promote the proliferation of EpH4-Ev cells, inhibit LPS-induced apoptosis, and the effect of MSC-ACE2 was stronger than these two.

### MSC-ACE2 Downregulates TLR4/MAPK/NF-κB Signaling Pathway Stimulation to Inhibit LPS-Induced Inflammation

**Figure 6:**
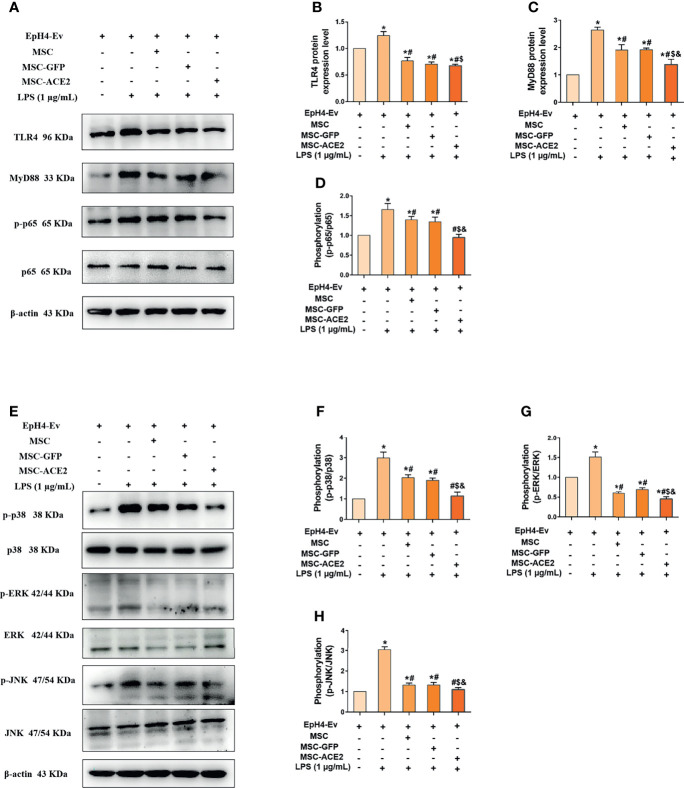
MSC-ACE2 downregulates TLR4/MAPK/NF-κB signaling pathway activation to inhibit LPS-induced inflammation in EpH4-Ev cells. **(A)** Detection of relative protein expression levels of TLR4, MyD88, p65 and phosphorylation levels of p65 by Western blot; **(B–D)** Statistics of TLR4, MyD88, p65 and phosphorylation levels of p65 proteins Western blot results; **(E)** Detection of relative protein expression levels of phosphorylation levels of p38, ERK, JNK, and p38, ERK, JNK by Western blot; **(F–H)**, Statistics of phosphorylation levels of p38, ERK, JNK, and p38, ERK, JNK Western blot results. Experiments were repeated three times and data were presented as the mean ± SEM (n = 4). *P < 0.05 vs. EpH4-Ev; ^#^ *P* < 0.05 vs. LPS; ^$^ *P* < 0.05 vs. MSC; ^&^ *P* < 0.05 vs. MSC-GFP.

We next explored the molecular signaling pathways through which MSC and MSC-ACE2 exert their anti-inflammatory effects. Western blot results (**Figure 6**) showed that LPS stimulation of EpH4-Ev cells significantly increased the protein expression levels of TLR4, its junction protein MyD88, phosphorylation levels of NF-κB (p65), p-p38, p-ERK and p-JNK compared with the control group (^*^ *P* < 0.05 *vs*. EpH4-Ev). Both co-cultured with MSC, MSC-GFP or MSC-ACE2 significantly inhibited the levels of TLR4, MyD88, phosphorylation levels of NF-κB (p65), p-p38, p-ERK, p-JNK (^#^ *P* < 0.05 vs. LPS) and suppressed the activation of TLR4/MAPK/NF-κB signaling pathway. The inhibition effect was stronger in MSC-ACE2 group over in MSC as well as MSC-GFP groups (^$^ *P* < 0.05 *vs*. MSC; ^&^ *P* < 0.05 *vs*. MSC-GFP). These results suggested that MSC-ACE2 can better inhibit the stimulation of TLR4/MAPK/NF-κB signaling pathway to resist LPS-induced inflammation in EpH4-Ev cells.

### MSC-ACE2 Upregulates IL-10/STAT3/SOCS3 Signaling Pathway Expression to Suppress LPS-Induced Inflammation in EpH4-Ev Cells

**Figure 7:**
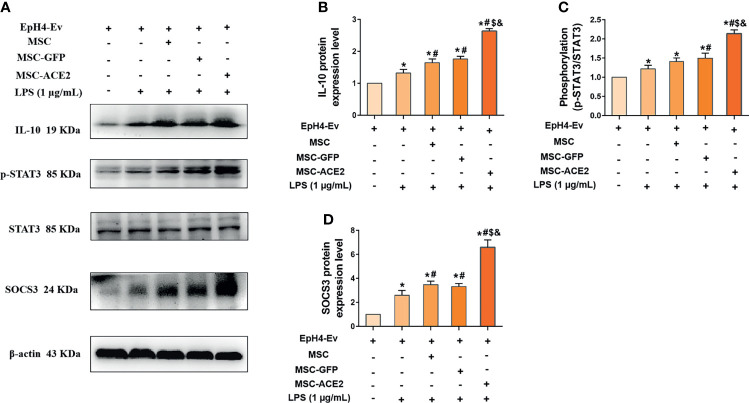
MSC-ACE2 upregulates IL-10/STAT3/SOCS3 signaling pathway expression to suppress LPS-induced inflammation in EpH4-Ev cells. **(A)** Detection of relative protein expression levels of IL-10, phosphorylation levels of STAT3, STAT3 and SOCS3 by Western blot; **(B–D)**, Statistics of the IL-10, phosphorylation levels of STAT3, STAT3 and SOCS3 Western blot results. Experiments were repeated three times and data were presented as the mean ± SEM (n = 4). ^*^ *P* < 0.05 vs. EpH4-Ev; ^#^ *P* < 0.05 vs. LPS; ^$^ *P* < 0.05 vs. MSC; ^&^ *P* < 0.05 vs. MSC-GFP.

The mRNA results (**Figure 7**) showed that MSC-ACE2 significantly increased the expression of IL-10, so we speculated whether MSC-ACE2 exerted anti-inflammatory effects through the IL-10/STAT3/SOCS3 signaling pathway. So we examined the expression of key proteins of the IL-10/STAT3/SOCS3 signaling pathway by Western blot. The results showed (**Figure 7**) that LPS stimulation of EpH4-Ev cells significantly increased the expression levels of IL-10, p-STAT3, SOCS3 compared with the control group (^*^ *P* < 0.05 *vs*. EpH4-Ev). Both co-cultured with MSC, MSC-GFP and MSC-ACE2 significantly increased the levels of IL-10, p-STAT3, SOCS3 (^#^ *P* < 0.05 *vs*. LPS), and activation of IL-10/STAT3/SOCS3 signaling pathway. The MSC-ACE2 group further increased the levels of IL-10, p-STAT3, SOCS3 more strongly compared to the MSC as well as MSC-GFP groups (^$^ *P* < 0.05 *vs*. MSC; ^&^ *P* < 0.05 *vs*. MSC-GFP). The outcomes suggested that MSC-ACE2 can better upregulation the expression of the IL-10/STAT3/SOCS3 signaling pathway to suppress LPS-induced inflammation in EpH4-Ev cells.

### MSC-ACE2 Upregulates Blood-Milk Barrier-Related Protein Expression to Suppress LPS-Induced Inflammation in EpH4-Ev Cells

**Figure 8:**
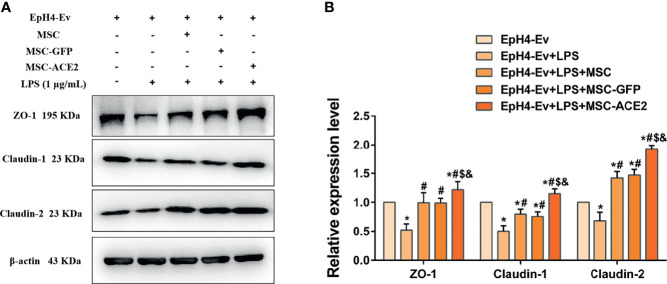
MSC-ACE2 upregulates blood-milk barrier-related protein expression to suppress LPS-induced inflammation in Eph4-EV cells. **(A)** Detection of relative protein expression levels of ZO-1, Claudin-1, Claudin-2 by Western blot; **(B)** Statistics of the ZO-1, Claudin-1, Claudin-2 Western blot results. Experiments were repeated three times and data were presented as the mean ± SEM (n = 4). ^*^ *P* < 0.05 vs. EpH4-Ev; ^#^ *P* < 0.05 vs. LPS; ^$^ *P* < 0.05 vs. MSC; ^&^ *P* < 0.05 vs. MSC-GFP.

Western blot results (**Figure 8**) showed that LPS treatment of EpH4-Ev cells resulted in significant downregulation of ZO-1, claudin-1 and claudin-2 protein expression levels (^*^ *P* < 0.05 *vs*. EpH4-Ev). However, co-cultured with MSC or MSC-GFP reversed the LPS-induced ZO-1, claudin-1 and claudin-2 protein expression down-regulation (^#^ *P* < 0.05 *vs*. LPS). Interestingly, this reversal was stronger for MSC-ACE2 group compared to MSC, and MSC-GFP groups (^$^ *P* < 0.05 *vs*. MSC; ^&^ *P* < 0.05 *vs*. MSC-GFP). These results suggested that LPS disrupted the blood-lactation barrier in EpH4-Ev cells, MSC repaired the blood-lactation barrier. So, MSC-ACE2 had a stronger repair effect.

## Discussion

**Figure 9:**
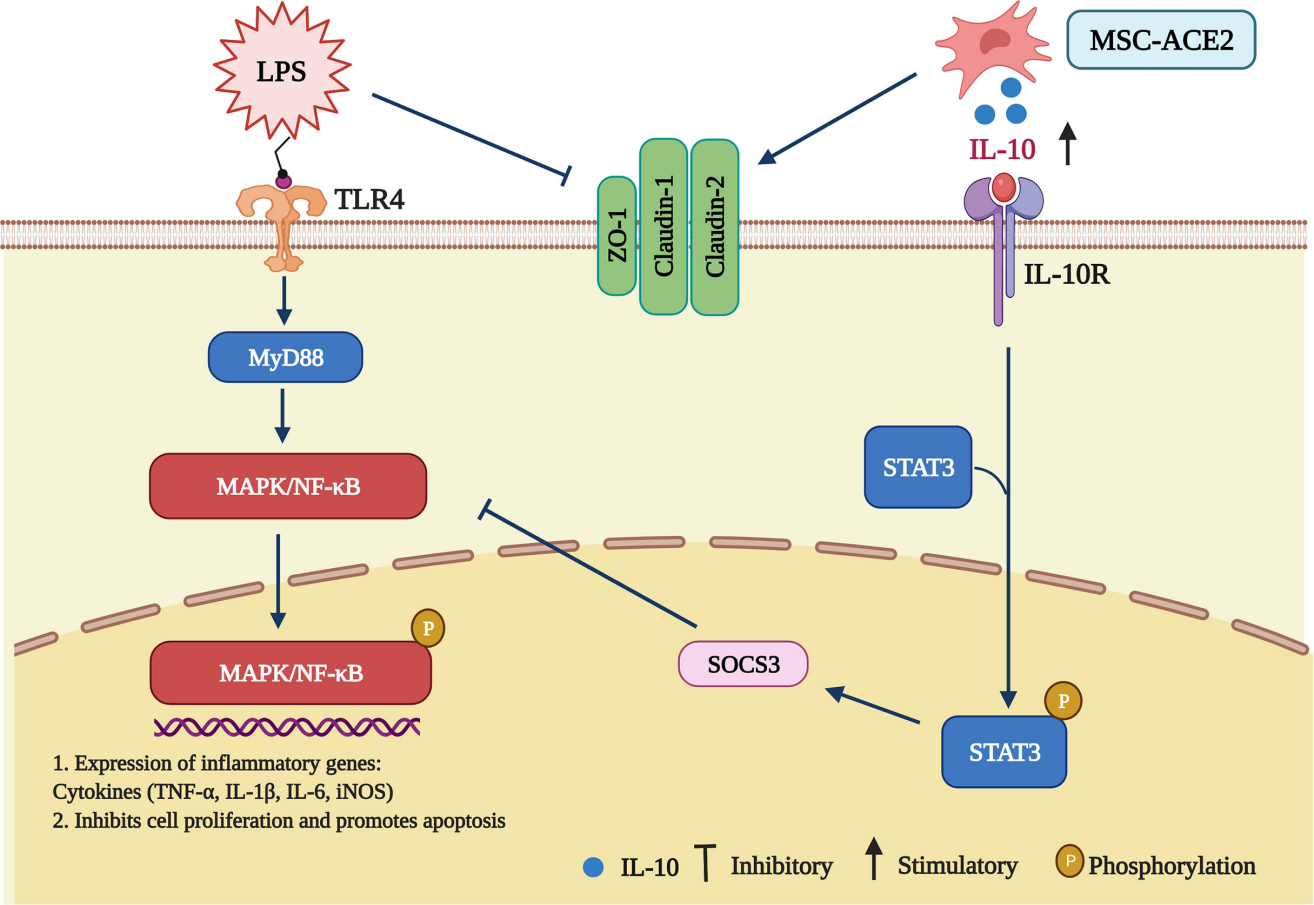
Schematic representation of MSCs overexpressing ACE2 favorably ameliorate LPS-induced inflammatory injury in mammary epithelial cells (Charting with BioRender.com software). 1 µg/mL of LPS concentration treated with EpH4-Ev for 9 h caused significant cell injury, activated MAPK/NF-κB signaling pathway, promoted the release of inflammatory factors TNF-α, IL-Iβ, IL-6, iNOS, inhibited cell proliferation, promoted apoptosis, disrupted blood-milk barrier and aggravated mammary epithelial cells injury. Co-culture with MSC, MSC-GFP or MSC-ACE2 reversed LPS-induced mammary epithelial cells injury, inhibited MAPK/NF-κB signaling pathway, activated IL-10/STAT3/SOCS3 signaling pathway, promoted cells proliferation, inhibited apoptosis, promoted tight junction protein expression and promoted blood-milk barrier repair, and MSC-ACE2 showed better anti-inflammatory and anti-injury effects than MSC and MSC-GFP.

Our study aimed to determine the therapeutic effect of MSC-ACE2 compared to MSC-GFP and MSC in LPS-induced injury in EpH4-Ev cells. The therapeutic potential of MSCs has been widely demonstrated in various diseases, including asthma (16), rheumatoid arthritis (17, 18), and Crohn’s disease (19, 20). Numerous clinical trials and tests around the world have validated the ability of MSCs to alleviate inflammation (15, 21, 22). In this study, we demonstrated that MSC and MSC-GFP can reverse LPS-induced injury in EpH4-Ev cells. The preventive result of MSC-ACE2 against EpH4-Ev cells injury, prompted by LPS that was more substantial over MSC or MSC-GFP therapy. We have demonstrated several mechanisms by which MSC-ACE2 exert synergistic therapeutic effects (**Figure 9**), including (1) a reduction in inflammatory cytokines, (2) an increase in cell proliferation, (3) a decrease in apoptosis, and (4) repair of a blood-milk barrier. These effects are mediated through down-regulation of TLR4/MAPK/NF-κB signaling pathway and up-regulation of the IL-10/STAT3/SOCS3 signaling pathway. Interestingly, it has been recently demonstrated that the TNF-TNFR2 signaling pathway controls the majority of MSC immunological and regenerative function (23, 24). Both ACE2 and TNFR2 have similar anti-inflammatory and anti-injury properties, so ACE2 and TNFR2 expression may have synergistic effects.

NAGase has been used as a marker enzyme to assess mammary epithelial cell damage, and infection with pathogenic bacteria *(E. coli*, *Streptococcus*, *Staphylococcus aureus*) or LPS stimulation can lead to increased mammary NAGase activity (25–28). Consistent with the expected results, MSC-ACE2 was found to significantly reverse the LPS-induced elevation of NAGase activity in this study, suggesting that MSC-ACE2 ameliorated LPS-induced injury to mammary epithelial cells. Present research has demonstrated that the renin-angiotensin system RAS, and its main biological effector Ang II, plays an important role in inflammatory diseases (29–31). Ang II is a strong pro-inflammatory mediator that exerts pro-inflammatory effects through the AT1 receptor (32). ACE2 hydrolyzes Ang II to generate Ang-(1-7) and exerts anti-inflammatory as well as anti-injury effects *via* the Mas receptor (33). Our study found that MSC-ACE2 alleviated inflammatory injury caused by LPS by downregulating Ang II. These results suggest that MSCs are a good ACE2 gene carrier and that the binding of MSCs to the ACE2 gene enhances the therapeutic effect of MSCs.

As a pluripotent stem cell, MSCs have a powerful immunomodulatory capacity to control the inflammatory response, alleviate and treat inflammatory diseases through surface molecules, secretion of soluble cytokines, etc. (34–38). Our results showed the anti-inflammatory impact of MSC-ACE2 is superior to that of MSC and MSC-GFP, which provides new ideas for the study of MSC and ACE2 in anti-inflammatory. Some scholars have already applied this new therapeutic idea in the lung (39–45) and kidney (36, 46). Gao et al. reported that MSC overexpressing ACE2 could treat bleomycin-induced acute lung injury in rats (39). He et al. found that MSC overexpressing ACE2 alleviated LPS-induced lung injury, and the mechanism of ACE2 overexpressed in the lung targeted Ang II for degradation, thereby alleviating lung endothelial injury (42). Liu et al. reported that ACE2-modified MSC significantly improved glomerular fibrosis in diabetic nephropathy (46). The above studies have mainly focused on lung and kidney, while the studies on animal mammary glands have not been reported yet, so this study is the first to report the role and mechanism of MSC-ACE2 in LPS-prompted inflammation in EpH4-Ev cells.

It is well known that MSC has powerful anti-inflammatory effects, while ACE2, a key enzyme of the RAS system, is well familiar for its powerful anti-inflammatory as well as anti-damaging effects. So the anti-inflammatory combine effects were greatly enhanced and significantly higher than those of MSC and MSC-GFP groups alone. To investigate the molecular mechanisms underlying the role of MSC-ACE2, we examined the expression of proteins related to cell proliferation and apoptosis pathways. The result showed that MSC-ACE2 promotes the regeneration of damaged cells by promoting the proliferation of EpH4-Ev cells and inhibiting their apoptosis, which is consistent with the previously reported mechanisms (15). LPS/MAPK/NF-κB, the classical signaling pathway of inflammation, is known for its ability to regulate inflammatory cytokines, transcription of cell proliferation, apoptosis, lymphocyte maturation, humoral immunity, innate immunity and immune organogenesis (47–49). The STAT3 (signal transducer and activator of transcription), a key transcriptional activator, prevents excessive TLR4-driven inflammatory responses, and also STAT3 pathway promotes IL-10 expression (50, 51). Our results suggest that MSC-ACE2 activates the IL-10/STAT3/SOCS3 signaling pathway while inhibiting the NF-κB and MAPK signaling pathways. The involvement of these two pathways further suggests that MSC-ACE2 can promote the secretion of IL-10 by EpH4-Ev cells through paracrine action to treat inflammation.

As the main component of the blood-milk barrier, mammary epithelial cells are an important line of defense for the mammary gland, and play an important role in defense against pathogenic microbial infections (52–54). Our study also found that LPS stimulation disrupted the blood-milk barrier of EpH4-Ev cells. While MSC, MSC-GFP, MSC-ACE2 repaired the blood-milk barrier, and also the repair effect of MSC-ACE2 was stronger than that of MSC as well as MSC-GFP alone. This is the first demonstration that MSC-ACE2 regulates the blood-milk barrier of mammary epithelial cells, providing a new therapeutic option for the future treatment of mastitis.

## Conclusions

Our results showed that ACE2-modified MSCs have stronger anti-inflammatory effects in LPS-induced EpH4-Ev cells injury and repaired the blood-milk barrier of EpH4-Ev cells injury as well as restored their cellular functions.

## Data Availability Statement

The original contributions presented in the study are included in the article/**Supplementary Material**. Further inquiries can be directed to the corresponding author.

## Author Contributions

Conceptualization, SY and YZ. Methodology, SY. Software, PY. Validation, SY, PY, and YZ. Formal analysis, MA. Investigation, XC. Resources, YZ. Data curation, NX. Writing-original draft preparation, SY. Writing-review and editing, SY. Visualization, MA. Supervision, PY. Project administration, YZ. Funding acquisition, YZ. All authors have read and agreed to the published version of the manuscript.

## Funding

This research was funded by the National Natural Science Foundation of China, grant number 31972640, and Priority Academic Program Development of Jiangsu Higher Education Institutions.

## Conflict of Interest

The authors declare that the research was conducted in the absence of any commercial or financial relationships that could be construed as a potential conflict of interest.

## Publisher’s Note

All claims expressed in this article are solely those of the authors and do not necessarily represent those of their affiliated organizations, or those of the publisher, the editors and the reviewers. Any product that may be evaluated in this article, or claim that may be made by its manufacturer, is not guaranteed or endorsed by the publisher.
