# Supplementary material for: Mesenchymal Stem Cells Overexpressing ACE2 Favorably Ameliorate LPS-Induced Inflammatory Injury in Mammary Epithelial Cells
Source: Front Immunol. 2022 Jan 14;12:796744. doi: 10.3389/fimmu.2021.796744 (PMC8795506; doi:10.3389/fimmu.2021.796744)
Supplement: Supplementary file 1 [file DataSheet_1.doc]

**Figure 1**


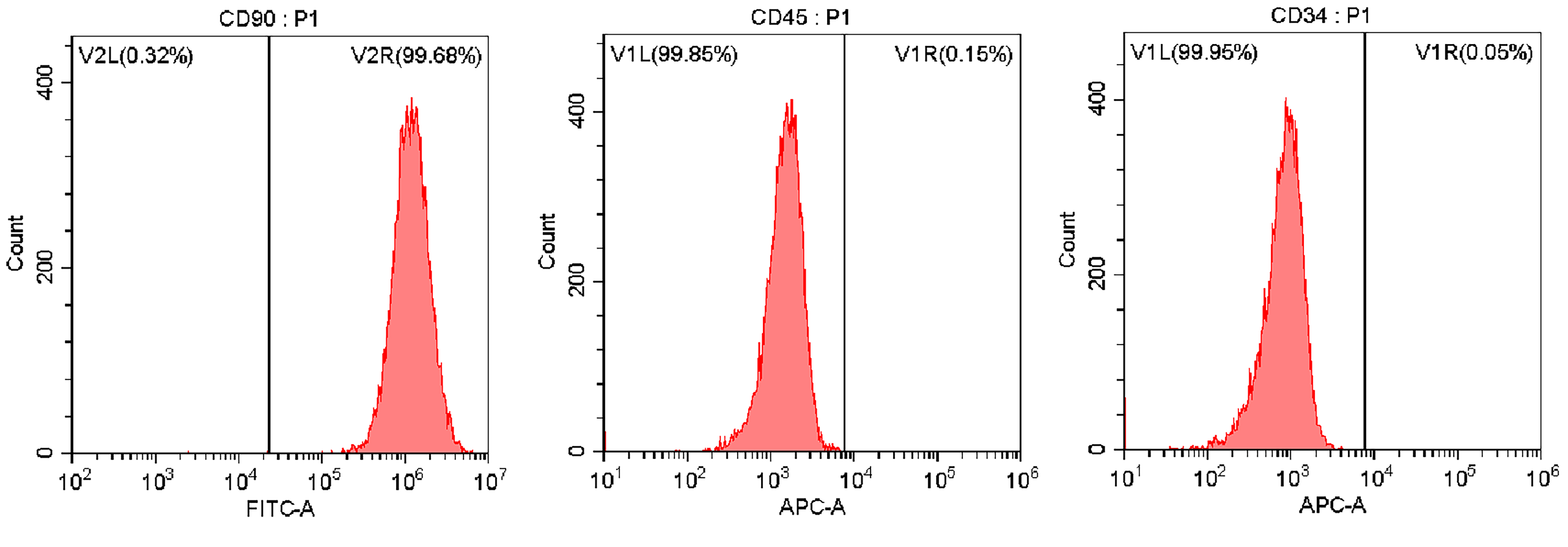


**Figure 1.** Third-generation MSCs were inoculated in 6-well plates and immunostained using CD90-FITC, CD45-APC, and CD34-APC antibodies (Biolegend, USA), with unstained MSCs serving as control. Analysis was performed in APC and FITC lanes using flow cytometry (Beckman Coulter Inc, Brea, CA, USA). Data were then analyzed using CytExpert software 2.0 (Beckman Coulter Inc, Brea, CA, USA).
