# Supplementary material for: Mesenchymal Stem Cells Overexpressing ACE2 Favorably Ameliorate LPS-Induced Inflammatory Injury in Mammary Epithelial Cells
Source: Front Immunol. 2022 Jan 14;12:796744. doi: 10.3389/fimmu.2021.796744 (PMC8795506; doi:10.3389/fimmu.2021.796744)
Supplement: Supplementary file 2 [file DataSheet_2.doc]

**Table S1** The primer sequences of the genes

| **Target genes** | **Primer sequences (5’-3’)** |
| --- | --- |
| TNF-α | TCCCAGGTTCTCTTCAAGGGA |
|  | GGTGAGGAGCACGTAGTCGG |
| IL-Iβ | GCCTCGTGCTGTCGGACCCATA |
|  | TGCAGGGTGGGTGTGCCGTCTT |
| IL-6 | CAAGAAAGACAAAGCCAGAGTC |
|  | GAAATTGGGGTAGGAAGGAC |
| IL-10 | CCAGGGAGATCCTTTGATGA |
|  | CATTCCCAGAGGAATTGCAT |
| iNOS | CACAGCAATATAGGCTCATCCA |
|  | GGATTTCAGCCTCATGGTAAAC |
| β-actin | TCTGGCACCACACCTTCTA |
|  | AGGCATACAGGGACAGCAC |
